# Retrospective Analysis of the Effectiveness of Remdesivir in COVID-19 Treatment during Periods Dominated by Delta and Omicron SARS-CoV-2 Variants in Clinical Settings

**DOI:** 10.3390/jcm12062371

**Published:** 2023-03-19

**Authors:** Krystyna Dobrowolska, Dorota Zarębska-Michaluk, Michał Brzdęk, Piotr Rzymski, Magdalena Rogalska, Anna Moniuszko-Malinowska, Dorota Kozielewicz, Marcin Hawro, Marta Rorat, Katarzyna Sikorska, Jerzy Jaroszewicz, Justyna Kowalska, Robert Flisiak

**Affiliations:** 1 Collegium Medicum, Jan Kochanowski University, 25-317 Kielce, Poland; 2 Department of Infectious Diseases and Allergology, Jan Kochanowski University, 25-317 Kielce, Poland; 3 Department of Infectious Diseases, Provincial Hospital, 25-317 Kielce, Poland; 4 Department of Environmental Medicine, Poznan University of Medical Sciences, 60-806 Poznań, Poland; 5 Department of Infectious Diseases and Hepatology, Medical University of Białystok, 15-540 Białystok, Poland; 6 Department of Infectious Diseases and Neuroinfections, Medical University of Białystok, 15-809 Białystok, Poland; 7 Department of Infectious Diseases and Hepatology, Faculty of Medicine, Collegium Medicum in Bydgoszcz, Nicolaus Copernicus University, 87-100 Torun, Poland; 8 Department of Infectious Diseases and Hepatology, Medical Center in Łańcut, 37-100 Łańcut, Poland; 9 Department of Forensic Medicine, Wrocław Medical University, 50-367 Wroclaw, Poland; 10 Institute of Maritime and Tropical Medicine, Faculty of Health Sciences, Medical University of Gdansk, 81-519 Gdynia, Poland; 11 Department of Infectious Diseases and Hepatology, Medical University of Silesia, 41-902 Katowice, Poland; 12 Department of Adults’ Infectious Diseases, Medical University of Warsaw, 01-201 Warsaw, Poland

**Keywords:** antiviral, pandemic, SARS-CoV-2, epidemiology

## Abstract

Continuous evaluation of real-world treatment effectiveness of COVID-19 medicines is required due to the ongoing evolution of SARS-CoV-2 and the possible emergence of resistance. Therefore, this study aimed to analyze, in a retrospective manner, the outcomes in patients hospitalized with COVID-19 during the pandemic waves dominated by Delta and Omicron variants and treated with remdesivir (RDV) (*n* = 762) in comparison to a demographically and clinically matched group not treated with any antivirals (*n* = 1060). A logistic regression analysis revealed that RDV treatment was associated with a significantly lower risk of death during both Delta wave (OR = 0.42, 95%CI: 0.29–0.60; *p* < 0.0001) and Omicron-dominated period (OR = 0.56, 95%CI: 0.35–0.92; *p* = 0.02). Moreover, RDV-treated groups were characterized by a lower percentage of patients requiring mechanical ventilation, but the difference was not statistically significant. This study is the first real-world evidence that RDV remains effective during the dominance of more pathogenic SARS-CoV-2 variants and those that cause a milder course of the disease, and continues to be an essential element of COVID-19 therapy.

## 1. Introduction

Severe acute respiratory syndrome coronavirus 2 (SARS-CoV-2), which causes Coronavirus disease 2019 (COVID-19), was first identified in Wuhan, China at the end of 2019. Due to the virus’s rapid spread, the World Health Organization (WHO) issued a pandemic declaration in mid-March 2020. At the same time, the scientific response to COVID-19 was unprecedented, with a high volume of research focusing on viral biology, diagnostics, clinical aspects of infection, preventive measures, and the development of vaccines and treatment options [1,2]. Since developing novel drugs against new viral diseases is time-consuming, the primary focus encompassed repurposed drugs [3,4]. However, prior to the COVID-19 pandemic, remdesivir (RDV), a non-canonical nucleotide, was developed as a broad-spectrum antiviral drug that terminates ribonucleic acid (RNA) replication by inhibition of the RNA-dependent RNA polymerase of RNA viruses of several families, including Coronaviridae, Paramyxoviridae, and Filoviridae [5,6]. Based on clinical evidence, RDV was authorized in June 2020 by the European Medicine Agency to treat COVID-19 in adults and adolescents (>12 years with weight ≥ 40 kg) who require oxygen therapy. It can also be used in adults who do not require oxygen supplementation but represent a high-risk group for severe COVID-19 [7].

Since RNA viruses are characterized by a very high mutation rate, SARS-CoV-2 is continuously subject to adaptive evolution [8]. This has resulted in the emergence of genetic variants that the WHO designated as variants of concern (VOCs) and which, through a comparative evaluation, have been found to reveal one or more of the following impacts at a degree of global public health significance: increased transmissibility, increased virulence, and decreased effectiveness of preventive and treatment measures, including vaccines and therapeutics [9]. So far, five lineages have been classified as VOCs: Alpha (B.1.1.7), Beta (B.1.351), Gamma (P.1), Delta (B.1.617.2), and Omicron (B.1.1.529). In Europe, three of them played a significant role in the COVID-19 pandemic: (i) Alpha, which was active in the first quarter of 2021, (ii) Delta, which dominated through June and December 2021 and (ii) Omicron, which has been the dominant lineage since early 2022 and continues to evolve with numerous subvariants identified.

The emergence and accumulation of nonsynonymous mutations have impacted, to a different degree, the effectiveness of several therapeutics, particularly anti-spike monoclonal antibodies [10]. However, antivirals that target other SARS-CoV-2 sites than the spike protein, including RDV, are less prone to be subject to resistance [11,12]. Nevertheless, the evolution of SARS-CoV-2 has also led to a rise of mutations in the sequence encoding RNA-dependent RNA polymerase (the structure of which is comprised of the viral proteins non-structural protein 12, and two accessory proteins, nsp8 and nsp7), targeted by RDV [13,14]. For example, a P314L mutation in nsp12, which co-occurred with the D614G mutation in spike protein, became widespread in mid-2020 [15]. Moreover, some mutations (e.g., P323L) were suggested to increase the viral mutation rate [16]. There is also experimental evidence that resistance to RDV could emerge in clinical settings and under RDV-selective pressure [17]. A single amino acid substitution in the polymerase (F548S) has been shown to be responsible for reduced susceptibility to remdesivir at low levels in Ebola virus infection treated with RDV, and given that remdesivir’s point of action is a structurally analogous region of the SARS-CoV-2 virus polymerase, molecular surveillance of this site in COVID-19 patients is recommended due to the possible risk of resistance [18]. Moreover, the emergence of de novo mutations of RDV resistance has been observed in treated immunocompromised patients [19,20,21]. There are different mechanisms through which the resistance to RDV may arise in SARS-CoV-2. For example, the S759A mutation was demonstrated to lead to a 10-fold decreased preference for RDV-triphosphate as a substrate, while V792I decreased the concentration of uridine triphosphate required to overcome the template-dependent association with RDV [17]. These findings stress the continuous need to monitor and compare the effectiveness of RDV against various SARS-CoV-2 variants. A number of clinical trials and real-world studies have confirmed the efficacy of RDV use in pandemic waves caused by previous variants of the virus, up to and including the Delta strain [22,23,24,25,26]. While there is in vitro evidence that RDV remains active against the Omicron variant at a level comparable to that against the Delta variant, confirmation of this in a clinical setting is scarce [27,28]. The emergence of spontaneous mutations in RNA-dependent RNA polymerase is even more plausible in the era of the Omicron lineage, as its escape from humoral immunity translated into a significant transmission advantage over the Delta variant, leading to increased risk of breakthrough infections and re-infections [29,30]. At the same time, Omicron lineage dominance overlapped with the period the pandemic restrictions were lifted, which may further contribute to its transmission, infections, mutations, and their spread.

This real-world study was designed to assess whether RDV treatment of patients hospitalized during the period of dominance of the Omicron variant retained the efficacy achieved during the dominance of the Delta variant. During both periods of the COVID-19 pandemic, the clinical course and outcomes of patients treated with RDV were compared with those not receiving any antiviral treatment.

## 2. Materials and Methods

### 2.1. Data Collection

The data for this study were retrospectively retrieved from SARSTer, a nationwide database managed by the Polish Association of Epidemiologists and Infectiologists, and used to collect observational data on patients hospitalized since the beginning of the COVID-19 pandemic. The project involved 44 Polish centers located in different regions of Poland.

The studied population was selected from the database of hospitalized adult patients infected with SARS-CoV-2. It included 1822 patients hospitalized during the following pandemic periods (i) from 1 August 2021 to 31 December 2021 (defined as Delta wave), and (ii) from 1 January 2022 to 30 April 2022 (defined as Omicron wave). Of these, 762 were treated with remdesivir (RDV), and the control group (NO AVT) included 1060 patients (680 during the Delta wave and 380 during the Omicron wave) who were not treated with RDV, or any other antiviral drug, and were matched by age, sex, body mass index (BMI), presence of any comorbidity, and oxygen saturation (SpO_2_) at admission. According to the product characteristics and recommendations, RDV was administered intravenously once daily for 5–10 days with a loading dose of 200 mg on day 1, followed by a maintenance dose of 100 mg to 762 patients (490 during the Delta wave and 272 during the Omicron wave) [31,32].

Similar to previous research from the SARSTer database [33,34], two periods of variants dominance were established based on sequences submitted by Polish laboratories according to the Global Initiative on Sharing All Influenza Data (GISAID), the most reliable database on SARS-CoV-2 variants prevalence in different regions of the world [35]. Infection of SARS-CoV-2 was diagnosed based on a positive polymerase chain reaction or antigen test result, while management and treatment followed current national recommendations for COVID-19 [31,32].

The patients’ demographic data included age, sex, BMI, and comorbidities. The laboratory data assessed at the baseline included C-reactive protein (CRP), procalcitonin (PCT), white blood cell (WBC) count, the absolute number of lymphocytes (ALC), neutrophils (ANC) and platelets (PLT), interleukin-6 (IL-6), D-dimer and alanine aminotransferase (ALT) activity. Upon admission to the hospital, patients were assigned to one of the categories based on the presence of symptoms and oxygen saturation (SpO_2_) when breathing room air; these comprised (1) asymptomatic, (2) stable symptomatic with SpO_2_ > 95, (3) unstable symptomatic with SpO_2_ 91–95%, and (4) unstable symptomatic with SpO_2_ ≤ 90, and acute respiratory distress syndrome (ARDS). The clinical course of the disease was assessed on admission to the hospital, and then after 7, 14, 21, and 28 days using an ordinal scale based on WHO recommendations, it was modified to the 8-point version to match the specificity of the Polish healthcare system and used in previous SARSTer research [36,37]. The score was defined as (1) not hospitalized, no activity restrictions; (2) not hospitalized, no activity restrictions and/or not requiring oxygen supplementation at home; (3) hospitalized, and not requiring oxygen supplementation and not requiring medical care; (4) hospitalized, not requiring oxygen supplementation, but requiring medical care; (5) hospitalized, requiring normal oxygen supplementation; (6) hospitalized, requiring non-invasive ventilation with high-flow oxygen equipment; (7) hospitalized, for invasive mechanical ventilation or extracorporeal membrane oxygenation, and (8) death. The collected data also included the use of medications during hospitalization, such as antivirals, immunomodulators, antibiotics, and low-molecular-weight heparin. Information on vaccination status and history of previous infections with SARS-CoV-2 was unavailable in the database.

Study endpoints were defined as the need for oxygen therapy, the need for mechanical ventilation, and 28-day mortality, and were compared between studied cohorts.

The study was conducted according to the guidelines of the Declaration of Helsinki. The SARSTer study had the approval of the Ethical Committee of the Medical University of Białystok (APK.002.303.2020). Patient consent was waived due to the retrospective design of the study.

### 2.2. Statistical Analysis

Statistical analyses were performed with Statistica v. 13 (StatSoft, Tulsa, OK, USA). Categorical data were described by frequencies and percentages. Group comparisons were performed using Pearson’s χ^2^ test. Continuous data (age, BMI, time of oxygenation, age of patients who died, and laboratory markers) were presented as means, standard deviations, and minimum and maximum values for some variables. Gaussian distribution was checked with the Shapiro–Wilk test. Differences between groups were assessed using the Mann–Whitney test for continuous, non-normally distributed variables, and Student’s t-test for variables with Gaussian distribution. Multiple logistic regression models were used to evaluate the association between RDV use and mortality of COVID-19 patients hospitalized during the Delta wave and Omicron-dominated period. The confounding variables included in each model were age > 70 years, obesity (BMI > 30 kg/m^2^), male sex, baseline SpO_2_ ≤ 90% at admission, and dexamethasone use. A *p*-value of less than 0.05 was considered statistically significant.

## 3. Results

### 3.1. Baseline Patients’ Characteristics

The number of patients analyzed during Delta predominance was 1170, of whom 490 received RDV treatment. During the Omicron surge, the number of patients enrolled in the study was 652, of whom 272 were treated with RDV. In both periods, a slight male predominance was observed. During the Omicron-dominated period, patients treated with RDV had significantly lower BMI and higher age than the corresponding group during the Delta wave (Table 1).

**Figure 1:**
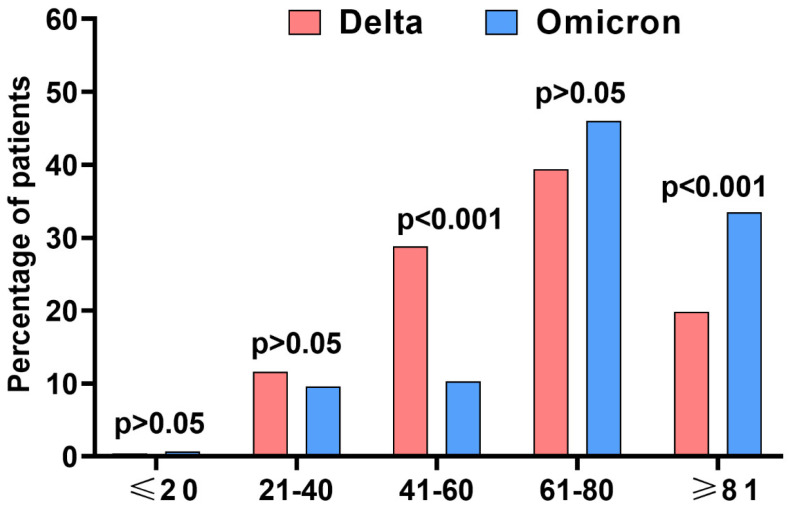
The age structure of patients treated with remdesivir during Delta wave (*n* = 490) and Omicron-dominated period (*n* = 272).

The largest RDV-treated group consisted of patients between 61 and 80 years of age, with those treated during the Delta variant predominance period being younger than Omicron variant-infected patients, who were significantly more likely to be over 80 years of age (Figure 1).

Comorbidities were significantly more frequently reported during the Omicron prevalence period (75.5 vs. 93%, *p* < 0.001), and these patients significantly more often presented with vascular diseases such as stroke (3.1 vs. 12.5%, *p* < 0.001), ischemic heart disease (9.4 vs. 23.5%, *p* < 0.001) (Table 1).

A comparison of the baseline clinical status of RDV-treated patients showed a significantly lower percentage of asymptomatic or in stable condition with saturation >95% (12% vs. 27.9%, *p* < 0.001) hospitalized during the Delta-dominant period, while the percentage of patients in unstable condition with saturation ≤95% or ARDS was significantly higher compared to the Omicron wave (87.9 vs. 72.1%, *p* < 0.001) (Table 2).

Despite the lack of differences in baseline demographic and clinical characteristics in both waves of the pandemic, patients treated with RDV during the Delta predominance period presented significantly lower mean white blood cell, neutrophil, and platelet counts and D-dimer concentrations, while during the Omicron predominance period they had lower platelets and IL-6 levels compared to those not receiving antiviral treatment (Table 2).

### 3.2. Remdesivir Therapy and Other Drugs in RDV-Treated Patients

Almost three-quarters of patients from the RDV-treated group received the drug for 5 days, in accordance with the summary of product characteristics. RDV was administered for over 5 days in six patients who were non-immunocompetent or had numerous risk factors for a severe course of the disease. The majority of patients treated with RDV in both waves of the pandemic received the drug within the first 5 days of symptom onset; the drug was first used earlier in the Omicron wave (3.5 ± 2.2 days) compared to in the Delta dominant period (5.1 ± 3.0 days; *p* < 0.001 (Table 3)).

The percentage of patients starting treatment within five days of symptom onset was 57.2% and 86.8% in the Delta and Omicron waves, respectively (*p* < 0.001), and more than half of the patients in the Omicron surge received therapy within the first three days compared to 27.8% in the Delta period (*p* = 0.001). Patients treated with RDV in the Delta wave were significantly more likely to require tocilizumab (*p* = 0.036), while dexamethasone was used in a comparative percentage of patients, and baricitinib, administered to single patients, was significantly more common in the Omicron wave (*p* = 0.047). Low molecular weight heparin at a prophylactic dose was used in a comparable percentage of patients in both waves, while the therapeutic dose was significantly more frequently received by patients in the Omicron wave.

### 3.3. Clinical Course and Outcomes of the Disease

#### 3.3.1. Comparison between RDV and No AVT Populations

**Figure 2:**
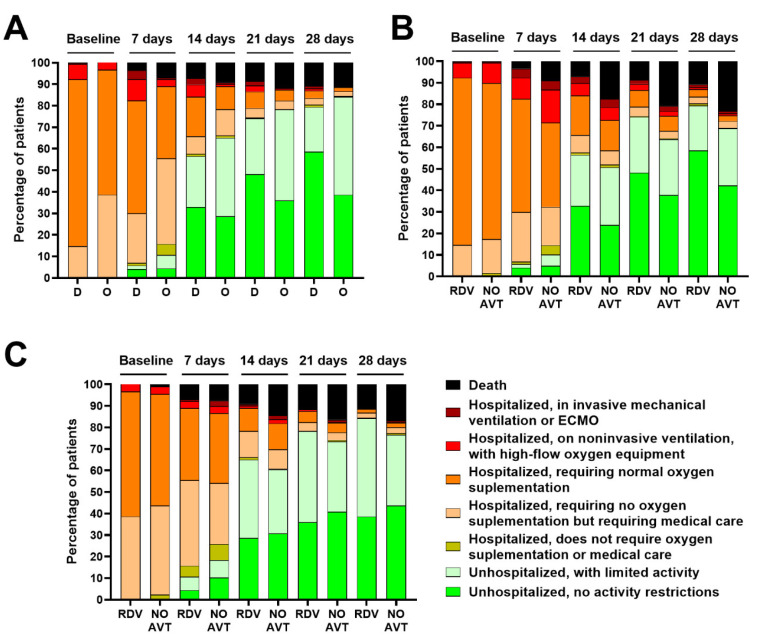
Ordinal scale categories at consecutive time points during Delta (D) and Omicron (O) surges in all patients treated with remdesivir (**A**), in all patients treated with remdesivir (RDV) or without antiviral treatment (NO AVT) during Delta (**B**) and Omicron (**C**) surges.

Comparing RDV-treated patients to those who did not receive antiviral therapy in the Delta period, we noted that antiviral therapy reduced hospitalization time in both waves of the pandemic (Figure 2).

Regardless of which variant of SARS-CoV-2 the patients were infected with, RDV therapy reduced the risk of death (Figure 2, Table 4).

The population of patients treated with RDV in the Omicron wave significantly more often required oxygen therapy during hospitalization compared to patients who did not receive antiviral therapy (*p* = 0.01). The opposite was observed in the Delta wave, but the difference was not statistically significant (Table 4). There were also no significant differences in the need for mechanical ventilation, although in both waves the percentage of patients requiring this management was lower among patients treated with RDV.

In both COVID-19 waves analyzed, the mortality rate of RDV-treated patients was lower compared to patients who did not receive the antiviral drug, with the difference being more than two-fold in the Delta wave, and statistically significant, and 1.5-fold in the Omicron wave without statistical significance. As shown by multiple logistic regression models, RDV treatment was an independent predictor of lower mortality of COVID-19 patients during both waves (Table 5). In addition, patients who received RDV treatment within 5 days of symptoms did not differ in mortality from those who received it later in the Delta wave (13.1 vs. 8.7%, *p* > 0.05) and Omicron-dominated period (11.2 vs. 14.3%, *p* > 0.05).

#### 3.3.2. Comparison of RDV-Treated Patients in Both Waves

The RDV-treated group during Delta and Omicron waves had comparable mortality and did not differ in the age of deceased patients, but the former required mechanical ventilation more often (Table 4).

The mortality rate of patients treated with RDV with baseline saturation ≤95% analyzed by age showed no difference between the two pandemic waves, while the need for mechanical ventilation was significantly more frequent in the Delta period, both overall and in the group of patients older than 60 years (Table 6).

This RDV-treated population also showed that drug administration within the first 5 days of symptom onset was associated with a significantly lower need for oxygen therapy in both waves compared to later initiation of treatment, while there was no effect of the timing of drug administration on the need for mechanical ventilation and mortality (Table 7).

Analyzing the effect of initiating RDV therapy with respect to the age of patients in the groups >60 and >80 years, no differences in the need for oxygen therapy, mechanical ventilation, or mortality were documented in the two waves between patients who received the drug within 5 days and those who started treatment more than 5 days after the onset of symptoms.

A comparison of patients treated with RDV in both waves of the pandemic showed that, at baseline and subsequent time points, patients infected with the Delta variant required oxygen supplementation or non-invasive ventilation more frequently, while patients admitted to the hospital during the Omicron wave were hospitalized for a shorter period. However, after hospital discharge, Omicron-infected patients were more likely to report activity limitations (20.8 vs. 45.6% on day 28) (Figure 2).

## 4. Discussion

RDV was the first antiviral drug shown to inhibit SARS-CoV-2 replication and receive an emergency use authorization, followed by registration for the treatment of patients with COVID-19. Its use was supported by the results of randomized clinical trials proving clinical improvement and shortening the recovery time and mortality rate in patients with mild to moderate course of infection [22,38]. Identifying highly infectious Omicron in late 2021 raised concerns about its impact on the pandemic dynamics and the effectiveness of the vaccines and COVID-19-approved therapies [39,40,41]. Based on literature reports of reduced activity of some monoclonal antibodies against this new lineage, some regulatory institutions, e.g., the U.S. Food and Drug Administration (FDA), limited their distribution [42]. At the same time, the available data indicated that antiviral drugs used in previous waves of the pandemic, including RDV, should remain active against the new SARS-CoV-2 variant [43]. However, such assumptions require direct evidence from clinical practice. Therefore, this real-world (RWE) study aimed to assess the effectiveness of RDV in a large cohort of Polish patients hospitalized with COVID-19 during the surges of two SARS-CoV-2 VOC, Delta, and Omicron.

As shown, under the dominance of both viral variants, RDV-treated patients were characterized by lower mortality than a group not treated with any antivirals, despite these groups being comparable in demographical and clinical parameters. During the Delta wave, the RDV-treated group had two-fold lower mortality. This finding clearly shows the benefits of RDV use during the dominance of this highly pathogenic SARS-CoV-2 variant [44]. However, the mortality in RDV-treated patients was also reduced, by approximately 1.5-fold, in the Omicron wave, which was generally characterized by a milder course of infection [33,34,45,46,47]. Multiple logistic regression clearly demonstrated that RDV use was an independent predictor of significantly decreased risk of death during the Delta wave and Omicron-dominated period.

These observations are consistent with results from clinical trials and RWE studies conducted in the early phases of the COVID-19 pandemic [22,48,49,50]. A retrospective analysis of a large U.S. patient cohort of nearly 100,000 patients hospitalized until February 2021 also confirmed a reduction in mortality in the overall population treated with RDV in absolute numbers (15.7 vs. 19.6%) but highlighted a significant difference only in patients who required low-flow oxygen therapy [51]. Another study, encompassing patients hospitalized until March 2021, indicated a statistically reduced mortality in women [52]. A study conducted in the Netherlands indicated that RDV use was associated with better outcomes during four pandemic waves, but the analysis did not cover the dominance of the Omicron variant [53]. To our knowledge, the present investigation is the first to address it in a real-world clinical setting.

Although the RDV-treated group was characterized by a lower percentage of patients requiring mechanical ventilation during both pandemic waves, the difference compared to a group not receiving any antiviral therapy was statistically insignificant. Nevertheless, the demonstration of a favorable trend in this regard is in line with reports by other authors [54,55]. Even in the Solidarity trial, the initial results of which were the basis for not recommending RDV in the WHO guidelines, the final report published in 2022 documented a significant beneficial effect on reducing mortality and disease progression requiring mechanical ventilation [56].

This analysis also shows that patients hospitalized due to COVID-19 during the Omicron wave were significantly older and more burdened with comorbidities, especially hypertension, ischemic heart disease, stroke, and neoplastic diseases. The clinical condition of those admitted to hospital during the Delta wave was more severe, as expressed in a significantly higher percentage of clinically unstable patients with oxygen saturation ≤95%. However, surprisingly, despite the more severe clinical condition, patients infected with Delta started antiviral therapy with RDV statistically later than patients infected with the Omicron variant. This could be due to the healthcare system being more overwhelmed during the Delta-dominated wave, delayed admission of patients to the hospital, and shortages of medicines, including RDV. The other possible explanation is related to the greater age and higher comorbidity burden of patients infected with the Omicron variant. Some of these patients could be admitted to the hospital for reasons other than COVID-19, underwent routine SARS-CoV-2 diagnostics during admission, tested positive on polymerase chain reaction or antigen test, and were immediately treated with RDV. Such phenomenon during the dominance of the Omicron variant had already been noticed in the United States [57].

In the current study, more than half of the Omicron-infected patients received RDV therapy within 3 days of the onset of symptoms, compared to nearly 28% in the Delta wave, and initiation of therapy within the first 5 days was documented in 87% and 57% of patients, respectively. Early drug administration has an impact on its effectiveness, and has been documented in clinical trials and numerous RWE analyses [22,48,52,58,59]. According to national recommendations, the optimal time to start RDV therapy is within the first 5 days; however, data from clinical trials have indicated the effectiveness of treatment starting up to 10 days from the onset of symptoms [22,32]. In this study, several patients started treatment with RDV even later than 5 days after the first symptoms. These were immunocompromised patients and those burdened with many risk factors for severe COVID-19, which justified such a decision by the attending physician [60].

Study limitations should be stressed. First, viral sequencing in hospitalized patients was not performed. However, the subdivision of Delta and Omicron-dominated periods was conducted based on reliable data deposited in GISAID. Second, the study did not include the immunization status of patients due to the unavailability of such data. Since immunization status is the result of both COVID-19 and vaccination, the history of infection, serological markers indicating previous contact with the virus, as well as the number of vaccine doses, would need to be taken into account, introducing further challenges to the analysis. With so many confounding factors, a simple distinction between vaccinated and unvaccinated individuals would not be reliable. Third, in some patients, especially during the Omicron wave, the diagnosis of COVID-19 could have been accidental due to the routine testing procedures of all patients admitted to the hospital. Fourth, the impact of other variables, such as the effectiveness of the health care system and environmental factors, including air pollution, which may have influenced patient mortality, was not analyzed [61]. Lastly, potential bias resulting from retrospective data collection based on medical records should be highlighted.

The main strength of this study was including and analyzing the data from a large real-world population from many different centers in our country, which ensured nationwide coverage and allowed the generalization of conclusions. Patients were managed according to the same national recommendations, and detailed medical records were available for each patient and information up to 28 days after admission unless death occurred earlier. In addition, to our knowledge, this is the first study that documents the effectiveness of RDV against the Omicron variant in a clinical setting and compares it with the period dominated by Delta SARS-CoV-2.

## 5. Conclusions

The present study shows that RDV remained an effective treatment of COVID-19 during Delta and Omicron SARS-CoV-2 waves. Under the dominance of both viral lineages, the use of RDV reduced the length of hospitalization, and mortality among hospitalized patients treated with RDV was significantly lower than among those who received no antiviral treatment, although the groups did not differ in demographic and clinical characteristics. Multiple logistic regression confirmed that RDV treatment was an independent predictor of a better outcome in COVID-19 patients during the dominance of both viral lineages. Despite the milder course of infections caused by the Omicron variant, RDV continues to be an important element of COVID-19 therapy. Further monitoring of RDV efficiency is encouraged as SARS-CoV-2 continues to evolve.

## Figures and Tables

**Table 1:** Baseline characteristics of patients with COVID-19 hospitalized during Delta and Omicron waves with regard to RDV treatment.

	Delta			Omicron			*p* (between RDV-Treated Patients in Delta and Omicron Wave)
	RDV (*n* = 490)	NO AVT (*n* = 680)	*p*	RDV (*n* = 272)	NO AVT (*n* = 380)	*p*	
Gender. females/males, %	47.1/52.9	46.5/53.5	>0.05	43.8/56.3	48.4/51.6	>0.05	>0.05
Age (years), mean ± SD	63.3 ± 17.1	65.1 ± 16.8	>0.05	70.6 ± 17.2	68.4 ± 18.8	>0.05	<0.001
BMI (kg/m^2^), mean ± SD	28.7 ± 5.1	28.2 ± 5.1	>0.05	27.2 ± 5.9	27.0 ± 5.3	>0.05	<0.001
SpO_2_	89.0 (6.7)	88.1 (8.1)	>0.05	91.7 (4.8)	91.6 (6.3)	>0.05	<0.001
Comorbidities, % (*n*)							
Any Comorbidity	75.5 (370)	77.2 (525)	>0.05	93.0 (253)	92.1 (350)	>0.05	<0.001
Hypertension	53.1 (260)	50.9 (346)	>0.05	60.7 (165)	52.9 (201)	0.049	0.04
Diabetes	21.4 (105)	19.7 (134)	>0.05	25.0 (68)	25.3 (96)	>0.05	>0.05
Stroke	3.1 (15)	6.2 (42)	0.015	12.5 (34)	9.5 (36)	>0.05	<0.001
COPD	4.5 (22)	5.3 (36)	>0.05	7.7 (21)	7.1 (27)	>0.05	>0.05
Neoplastic diseases	7.6 (37)	6.5 (44)	>0.05	13.2 (36)	16.3 (62)	>0.05	0.0106
Ischemic heart diseases	9.4 (46)	12.6 (86)	>0.05	23.5 (64)	18.9 (72)	>0.05	<0.001
Other CVD	19.2 (94)	20.7 (141)	>0.05	33.1 (90)	31.8 (121)	>0.05	<0.001
Other respiratory diseases	8.2 (40)	7.5 (51)	>0.05	11.8 (32)	9.5 (36)	>0.05	>0.05
Other metabolic diseases	10.6 (52)	11.3 (77)	>0.05	16.2 (44)	13.7 (52)	>0.05	0.03
Others	44.5 (218)	47.6 (324)	>0.05	72.8 (198)	70.8 (269)	>0.05	<0.001

AVT, antiviral therapy; BMI, body mass index; COPD, chronic obstructive pulmonary disease; CVD, chronic vascular disease; RDV, remdesivir; SD, standard deviation; SpO_2_, saturation of peripheral oxygen.

**Table 2:** Baseline clinical status and laboratory parameters (mean ± SD) of patients hospitalized during Delta and Omicron waves with regard to remdesivir (RDV) treatment.

	Delta			Omicron			*p* (between RDV-Treated Patients in Delta and Omicron Wave)
	RDV (*n* = 490)	NO AVT (*n* = 680)	*p*	RDV (*n* = 272)	NO AVT (*n* = 380)	***p***	
CRP, mg/L	82.5 ± 71.7	94.0 ± 84.5	>0.05	67.6 ± 67.6	69.9 ± 78.9	>0.05	<0.001
PCT, ng/mL	0.4 ± 1.3	1.1 ± 8.3	>0.05	0.9 ± 3.1	1.8 ± 9.2	>0.05	>0.05
WBC, ×10^3^/µL	7.0 ± 6.5	7.1 ± 3.9	0.006	7.8 ± 8.0	8.0 ± 4.9	>0.05	0.003
Lymphocytes, ×10^3^/µL	1.4 ± 4.7	1.1 ± 1.4	>0.05	1.3 ± 1.8	1.2 ± 1.5	>0.05	0.01
Neutrophils, ×10^3^/µL	5 ± 3.4	5.3 ± 3.2	0.008	5.5 ± 4.7	6.1 ± 6.0	>0.05	>0.05
Platelets, ×10^3^/µL	185.5 ± 79.5	213.0 ± 96.0	<0.001	202.4 ± 93.2	212.8 ± 101.5	0.04	0.006
IL-6, pg/mL	102.9 ± 329.1	122.4 ± 383.1	>0.05	160.3 ± 611.8	218.2 ± 1399.5	0.006	>0.05
d-dimer, ng/mL	1904.9 ± 5909.8	2165.4 ± 4917.6	0.004	2282.4 ± 4918.7	2809.6 ± 8179.5	>0.05	0.03
ALT, IU/L	42.5 ± 40.6	47.8 ± 51.1	>0.05	34.9 ± 31.6	45.3 ± 96.3	>0.05	<0.001
Stable symptomatic, SpO_2_ > 95% or asymptomatic	12.0 (59)	14.3 (97)	>0.05	27.9 (76)	34.7 (132)	>0.05	<0.001
Unstable symptomatic, SpO_2_ ≤ 95% or ARDS	87.9 (431)	85.9 (579)	>0.05	72.1 (196)	65.5 (248)	>0.05	<0.001

Abbreviations: AVT, antiviral therapy; ALT, alanine aminotransferase; ARDS, acute respiratory distress syndrome; CRP, C-reactive protein; IL-6, interleukin-6; PCT, procalcitonin; RDV, remdesivir; SD, standard deviation; SpO_2_, saturation of peripheral oxygen; WBC, white blood cells.

**Table 3:** Remdesivir therapy and other drugs used in remdesivir-treated patients during Delta and Omicron waves.

Parameters	Delta (*n* = 490)	Omicron (*n* = 272)	*p*
Time between onset of symptoms and start of the antiviral treatment, mean ± SD (min–max)	5.1 ± 3.0 (0–21) *n* = 479	3.5 ± 2.2 (0–14) *n* = 266	<0.001
Patient treated within 5 days of symptoms, % (*n*)	57.2 (274/479)	86.8 (231/266)	<0.001
Patient treated within 3 days of symptoms, % (*n*)	27.8 (133/479)	53.4 (142/266)	<0.001
Immunomodulators, % (*n*)	56.3 (276)	55.9 (152)	>0.05
Tocilizumab	14.9 (73)	9.6 (26)	0.04
Dexamethason	49.6 (243)	54.4 (148)	>0.05
Baricitinib	1.2 (6)	3.3 (9)	0.04
Antibiotics, % (*n*)	34.1 (167)	41.2 (112)	0.05
Low molecular weight heparin in prophylactic dose, % (*n*)	73.1 (358)	73.5 (200)	>0.05
Low molecular weight heparin in a therapeutic dose, % (*n*)	16.9 (83)	23.5 (64)	0.03

Abbreviations: SD, standard deviation.

**Table 4:** Endpoints of hospitalized patients during Delta and Omicron waves with regard to remdesivir (RDV) treatment.

	Delta			Omicron		
	RDV (*n* = 490)	NO AVT (*n* = 680)	*p*	RDV (*n* = 272)	NO AVT (*n* = 380)	*p*
Need for oxygen therapy, % (*n*)	75.9 (372)	77.1 (524)	>0.05	59.6 (162)	50.3 (191)	0.01
Need for mechanical ventilation, % (*n*)	7.8 (38)	9.4 (63)	>0.05	2.2 (6)	4.0 (15)	>0.05
Mortality, % (*n*)	10.8 (53)	23.2 (158)	<0.001	11.4 (31)	16.8 (64)	0.05
Age of patients who died (years), mean ± SD (min–max)	75.6 ± 13.9 (34–95)	76.5 ± 12.9 (30–99)	>0.05	77.7 ± 12.4 (37–95)	79.0 ± 14.2 (25–99)	>0.05

Abbreviations: AVT, antiviral therapy; RDV, remdesivir; SD, standard deviation.

**Table 5:** Multiple logistic regression on the association between mortality of COVID-19 patients during Delta and Omicron waves and selected variables, including remdesivir treatment (RDV).

Variable	Delta	Omicron
	Odds Ratio (95% Confidence Interval)	
Age >70 years	4.99 (3.55–7.01) *p* < 0.0001	2.2 (1.9–5.8) *p* < 0.0001
Male sex	0.82 (0.66–1.28) *p* > 0.05	0.71 (0.44–1.13) p > 0.05
Obesity (BMI > 30 kg/m^2^)	0.82 (0.56–1.20) *p* > 0.03	1.40 (0.92–1.89) *p* = 0.02
SpO_2_ ≤ 90% at admission	1.37 (0.89–4.63) *p* < 0.0001	2.40 (1.47–3.91) *p* = 0.0005
Dexamethasone treatment	1.81 (1.23–2.67) *p* = 0.003	2.97 (1.81–4.89) *p* < 0.0001
RDV treatment	0.42 (0.29–0.60) *p* < 0.0001	0.56 (0.35–0.92) *p* = 0.02

Abbreviations: BMI, body mass index; SpO_2_, saturation of peripheral oxygen; RDV, remdesivir.

**Table 6:** Twenty-eight-day mortality and the need for mechanical ventilation in patients treated with remdesivir during periods of Delta and Omicron variant dominance analyzed in all patients, only those with oxygen saturation ≤95%, including administration of remdesivir within 5 days of symptom onset and those aged over 60 or 80 years.

Patients Subpopulations	Mortality			Mechanical Ventilation		
	Delta	Omicron	*p*	Delta	Omicron	*p*
All patients, % (*n*/N)	10.8 (53/490)	11.4 (31/272)	>0.05	7.8 (38/490)	2.2 (6/272)	0.002
SpO_2_ ≤ 95%, % (*n*/N)	11.8 (51/431)	14.3 (28/196)	>0.05	7.9 (34/431)	3.1 (6/196)	0.02
SpO_2_ ≤ 95%, 0–5 days, % (*n*/N)	13.3 (31/233)	14.3 (23/161)	>0.05	7.7 (18/233)	2.5 (4/161)	0.03
SpO_2_ ≤ 95%, 0–5 days, >60 years, % (*n*/N)	19.5 (30/154)	15.4 (22/143)	>0.05	9.1 (14/154)	2.1 (3/143)	0.01
SpO_2_ ≤ 95%, 0–5 days, >80 years, % (*n*/N)	35.3 (18/51)	21.4 (12/56)	>0.05	7.8 (4/51)	1.8 (1/56)	>0.05

Abbreviations: SpO_2_, saturation of peripheral oxygen.

**Table 7:** Endpoints considering the time of RDV treatment initiation: within 5 days of symptom onset versus more than 5 days.

Parameter	Delta			Omicron		
	≤5	>5	*p*	≤5	>5	*p*
All						
*n*	274	205		231	35	
Mortality, % (*n*)	13.5 (37/274)	7.8 (16/205)	>0.05	10.8 (25/231)	14.3 (5/35)	>0.05
Need for oxygen therapy, % (*n*)	73 (200/274)	83.9 (172/205)	<0.001	56.7 (131/231)	77.1 (27/35)	0.0218
Time of oxygen therapy, mean ± SD	8.4 ± 9.4	10.1 ± 8.7	>0.05	9.4 ± 7.0	11.2 ± 6.8	>0.05
Need for mechanical ventilation, % (*n*)	8.8 (24/274)	6.8 (14/205)	>0.05	1.7 (4/231)	5.7 (2/231)	>0.05
>60 years						
*n*	172	110		182	29	
Mortality, % (*n*)	20.3 (35/172)	10 (11/110)	>0.05	12.6 (23/182)	17.2 (5/29)	>0.05
Need for oxygen therapy, % (*n*)	79.1 (136/172)	27.3 (30/110)	>0.05	63.7 (116/182)	75.9 (22/29)	>0.05
Time of oxygen therapy, mean ± SD	9.6 ± 9.9	10.7 ± 9.1	>0.05	9.3 ± 7	10.3 ± 6.7	>0.05
Need for mechanical ventilation, % (*n*)	9.9 (17/172)	7.3 (8/110)	>0.05	1.6 (3/182)	0 (0/29)	>0.05
>80 years						
*n*	56	36		75	13	
Mortality, % (*n*)	33.9 (19/56)	16.7 (6/36)	>0.05	16 (12/75)	30.8 (4/13)	>0.05
Need for oxygen therapy, % (*n*)	89.3 (50/56)	83.3 (30/36)	>0.05	62.7 (47/75)	84.6 (11/13)	>0.05
Time of oxygen therapy, mean ± SD	10.1 ± 8.9	10.9 ± 8.1	>0.05	8.4 ± 5.6	11.7 ± 7.4	>0.05
Need for mechanical ventilation, % (*n*)	8.9 (5/56)	8.3 (3/36)	>0.05	1.3 (1/75)	0 (0/13)	>0.05

Abbreviations: RDV, remdesivir; SD, standard deviation.

## Data Availability

Data supporting reported results can be provided upon request from the corresponding author.
